# Miscellanies

**Published:** 1837-01-01

**Authors:** 


### MISCELLANIES.

### Hahnemania.

A Practical View of Homœopathy, &c. By Stephen Simpson, M.D.

By this the latest and best work on homœopathy, we learn that Hahnemann was little better than a madman, and hence we are authorized in the title which we give to this short article.

"The writer strongly protests against the denomination of homœopathist, but still more strongly against the imputation of Hahnemannian homœopathism, being firmly of opinion that Homœopathy, as it has recently been taught and practised by its great discoverer, is little better than a mere chimera."

Again—

"In the mean time, the writer unhesitatingly declares, that he holds the hypotheses of Hahnemann as altogether unsatisfactory, his practical rules deduced from them as contrary to experience, and his theory of Chronic Diseases as devoid of all proof; of which last indeed it has been very justly observed that 'what is in it is true, is not new, and what is new, is not true.'"

Still farther on—

"Many, indeed, of the disciples of Hahnemann, out of a laudable regard for the feelings of their venerable teacher, now in his eighty-second year, hesitate to pronounce their recantation of the errors into which he has unnecessarily led them by his too hasty deductions."*

* Simpson.

What say the Hahnemaniacs and Hahnemuneale to this statement?—Here is the god of their idolatry at once deposed, and his empire—a small German principality it is true—usurped by one of his own disciples! But then, from the ashes of the chimera a phœnix is to rise, whose wings shall overspread the medical horizon, and cast in the shade all the systems and experience of former ages. We shall not waste much time on this system; but we may observe that, granting, for the sake of argument, that every part of this system of homœopathy was as true as Holy Writ, both in its principles and details, yet we assert, and hope to prove, from Dr. Simpson's own statements, that it is utterly incapable of being brought into practice among the profession of this or any other country in the world. It is only adapted for a few of the dilletanti who may choose to diddle dukes and humbug hypochondriacs—or for the dreams of enthusiasts, such as Hahnemann, and the respectable portion of his followers—amongst whom we have no hesitation in ranking Dr. Simpson and the few who have openly embraced homœopathy in this metropolis. But looking at the new system in all its bearings, and without the slightest prejudice or jealousy, we do believe that it opens the widest door to quackery and knavery that has ever yet been presented to the adventurous and unprincipled in our profession. This by the way. We now proceed to shew its impracticability in general.

Homœopathy must be learnt by practising on the healthy subject. "The individuals upon whom the trials are to be made, must be selected from both sexes and of different ages." "They must be in *perfect health.*" "Every slight deviation from healthy action in the functions of the internal organs must render all experiments more or less liable to error." Then the diet must be most systematic. "All fermented liquors, coffee, and even tea, if possible, spices of every description, all green vegetables and roots, excepting green peas, kidney-beans, carrots, cauliflower and potatoes, &c. must be altogether avoided." And all this by people in health, the subjects of experiments!! But not only must multitudes of all ages and both sexes become the recipients of homœopathic remedies, when they are in perfect health, they must also be free from all over-exertion of mind or body—all free indulgence of the passions—there must be "no pressing affairs to divert the mind from an accurate analysis of the internal sensations of the body." The experiments must be "repeated in the *same individuals* under various modifications of it (diet) so as to determine the influence of circumstances, &c." Then, again, the medicines must be tried in large, small, and infinitesimal doses, still on multitudes of healthy people. "Not only must the symptoms themselves be taken down, but also the peculiar circumstances by which they may be in any way modified; whether as to the influence of position, of movement, of rest, of confined or open air, of atmospheric changes, of day or night, of eating and drinking, constitutional differences, &c."

The above are only a few of the labours and difficulties attendant on the study of homœopathy! We thought that, by abjuring anatomy, pathology, &c., the Hahnemaniac practice would be rendered simple and easy, as compared with that of allopathy. But no. The preliminary study of homœopathy, on the healthy population alone, would require half a century at least—and *then* the student would just be ready to commence attendance on the sick—when about the age of 70 years! Is it worth while to pursue the subject farther? We conscientiously think it is not. The Hahnemania, like other manias, will run its day, and vanish into thin air—though not before many lives are sacrificed at the altar of this new illusion. We may just hint, however, that from some facts which have come to our knowledge, there is a strong under-current of self-interest—perhaps something that deserves a harsher term—in HAHNEMANIA. For a long time past, it is well known that there has existed in the public mind, a strong prejudice against polypharmacy—or rather against the large quantities of medicine which the general practitioner is obliged to prescribe, in self-defence against a villainous system of remuneration. Here, then, is an excellent opening for a host of homœopathic *conformers* to break loose upon the public with the cry of war upon large doses of medicine, and *vivas* to the Hahnemaniac system of curing diseases with the decillionth part of a grain or drop of physic! If it be said that this is an ungenerous insinuation, we reply, look at the crowded state of the profession, and say whether there will not be found a certain portion of them, like Shakespeare's poor *apothecary*, whose necessities will force *them* to adopt means of subsistence which their consciences cannot approve?

One word before we part from Dr. Simpson. He has illustrated his system of Hahnemania by the detail of thirty-two cases. How many of these, gentle reader, do you suppose are from his own experience? Twenty? No. Fifteen? No. Ten? No. Five? No. ONE SINGLE CASE is adduced by the author of a new system, as a specimen of its working!! Thirty-one cases are raked up from the Hahnemaniac journals of Germany—where no detection of error or misrepresentation can be put in force! For our own parts, we put full as much credence in the wildest of Baron Munchausen's stories, as in the soberest of these German reveries. The slender and solitary basis which Dr. Simpson has given us from his own note-book, we shall here insert. Even this one case will give rise to reflections not very favourable to homœopathy?

"M. N***, a young gentleman, about six years of age residing in London, was attacked in June last by slight catarrhal symptoms, which readily gave way to the exhibition of two or three doses of chamomile. After the cough had altogether subsided, however, there remained a peculiar tone or flatness of the voice which induced me to believe that some mischief was going on in the windpipe, though at the time no other symptoms could be detected. I, therefore, directed the friends to let me know immediately should a return of the cough take place. In the afternoon of the same day, I received a note stating, that since dinner the little patient had been attacked by severe fever, and a particularly harsh and distressing cough: he also complained of pain about the windpipe. Having sent a dose of aconite 6, I determined to visit the child myself in the evening; but, before I could reach the house about nine o'clock, another messenger had been dispatched to say that the child was much worse and was threatened with suffocation: the nurse too had immediately recognized the disease as the croup, having some time previously attended a child labouring under that affection; and indeed, the character of the disease could not be mistaken, even without entering the room, by any one who had once heard a croupy cough. Since seven o'clock, the little patient had had several fits of coughing with great straining and occasional retching: *he was now lying in the most anxious disquiet, in a burning fever with a dry skin, an extremely frequent and quick pulse, a hurried and wheezing respiration with clangous tone and occasional fits of a shrill and suffocative cough, which prevented all repose; the voice too was scarcely audible, and there was tenderness of the larynx upon pressure.*

From the preceding catarrhal symptoms, the tenderness of the larynx, and the intensity of the fever, there could be no doubt of the inflammatory character of the disease, and hence the importance of arresting the inflammation before exudation should take place over the lining membrane of the windpipe. For this purpose aconite and spongia are the best known specifics. The first dose of aconite having been given about an hour previously, a second was now exhibited, and arrangements made to repeat it alternately with spongia 3, every hour till the symptoms should subside. About twelve o'clock, it was evident that a considerable amelioration had taken place, *as the skin was covered with a profuse perspiration,* and the cough and difficulty of breathing were less severe, so that though still restless, the little patient was able to get some sleep at intervals. The medicines were now ordered to be given every two hours. In this way the improvement proceeded very gradually till seven o'clock in the morning, when the child fell into a sound sleep, and awoke about ten o'clock perfectly free from fever with a soft and moist skin; the cough too had become quite loose and entirely lost its croupy tone, and the voice was nearly natural, though still a little hoarse. As the bowels were much constipated, a dose of nux vom. 12, was given at night with the desired effect, and subsequently a dose or two of drosera 3, to remove the slight remaining hoarseness and cough, with which the treatment was concluded and the health of the patient rapidly restored."

We have marked a passage in italics, and we ask any man who has had the least experience in croup, whether he would trust to aconite and sponge under the pressure of such symptoms as are there described? Fortunately for homœopathy, a profuse perspiration saved the life of the child and the credit of the system. Had the case proved fatal, would it have been recorded in the annals of Hahnemania?

Now Dr. Simpson either has had, or has not had personal experience in the practice of homœopathy. If he *has* had personal observation, why has he not illustrated the system from his own practice? If he has only had *one* case, how can he so confidently recommend the new doctrine? Utrum horum? But we dismiss the subject, because it is a very unprofitable one.

---

### How to make Stays.

In our last number we noticed a very clever little book, by Mr. Coulson, on Deformities of the Chest. That book has arrived at a second edition, which lies at this instant before us. Amongst other things, it contains a chapter on stays, which is really both amusing and instructive. We would lay a bet that there is scarcely a surgeon in this country who knows the construction of the best modern stays. Yet they exercise a great influence on the figure—are of consequence in the treatment as well as prevention of deformities—and ought to be understood by all who may be called upon to treat such disorders. Mr. Coulson has seen things in the right light, and taken lessons in stay-making of Mrs. N. Geary, whom every body knows. Under these circumstances, it would be unpardonable, if we did not put our readers in the way of giving their fair patients a hint. And, if Sterne found that there were worse occupations than feeling a pretty woman's pulse, doctors may discover that there are less pleasant duties than lacing a lady's corset. But mind what you are about, *young* gentlemen—that's all. We are philosophers, and handle a "busk" or a "gore," without an idea of danger.

To proceed methodically:—There are, of course, three sorts of corset. 1. The corset for growing girls. 2. The demi-corset for the morning. 3. The complete corset. The pressure of the first is slight; being elastic in front, it yields to all the motions of the body, and when made with long and very elastic shoulder-straps, it allows perfect freedom of motion for exercise.

*Demi-corsets for the morning* are made about eight or ten inches in height, furnished here and there with light whalebones. In other respects, they are of the form of the upper part of the common corset; but the back edge ends in two long flaps, which are fastened in front by means of a tape. All this, however, is child's play. It is in the complete corset that the genius of Mrs. Geary has had full swing.

The problem to be solved was to support the figure, yet not to diminish freedom of motion—and to conceal the size of the abdomen, when too large.

"A little consideration of the shape will show that, in the back of the stays, extension throughout is chiefly wanted; in the front, extension throughout, and pressure inferiorly perhaps; and on the sides, extension throughout, slight pressure or support above, toward the bosom perhaps, and adaptation or pliability chiefly in the middle and below.

The extension throughout the back should be produced by two pliant whalebones, or, in some cases perhaps, by two thin steels.

The extension throughout the front, and the pressure, if necessary, to repress any prominence inferiorly, should be produced by a tempered steel of about an inch and a half wide, bent inward in a semicircular form, and sufficiently long to extend over the prominence.

The whole front, however, should be capable of expansion horizontally or laterally, by a portion of it, corresponding to the busk, placed over it, and like it extending from the top to the bottom of the front, being formed of numerous transverse elastics, while the lining behind the busk is divided longitudinally; so that this structure expands, in the transverse direction, with every flexure of the body.

Persons whose breasts are close may wear, inside the corset, at the upper extremity of the pocket of the busk, a piece of cotton wadding covered with white kid, to prevent the disagreeble chafing which the corners of the busk may produce: but it must be remembered that this adds to the thickness, and consequently to the pressure, and it should not, therefore, be unnecessarily or carelessly done. At the other end of the busk, may be placed a flat wadding.

The extension, support and pliability on the sides, may have somewhat varied means, according to the form of the individual."

To support the breasts, the "gores" are made too short in general, and the bosom is eventually deformed, and marked with long white streaks. Now see what the talent of Mrs. Geary has done.

"With regard to these corsets, Mrs. N. Geary has introduced many remarkable reforms. They are now made lighter than they were lately; and the bosom is so formed as no longer to press injuriously upon the breast or to form a crease above it. The formation of the bosom, indeed, and of all parts adapted to prominences, in Mrs. N. Geary's stays, is a matter of great ingenuity, and of extraordinary beauty. To this she was led by a desire to obviate the perpetual and just complaints made by ladies of the tightness across the chest, just below the bosom gores, in the common stays. To obviate these, she forms the whole stay of some nineteen or twenty longitudinal portions, or portions extending from the top to the bottom of the stays. Each of these constituent portions is wider above or below, than it is in the middle. By means of this gradually increasing width, the bosom and other prominent parts are formed. A moment's reflection will show that, by varying the width of any one or two of these portions, such stays become capable of adaptation to every possible variety of form; and that the bosom and all the prominent parts are thus formed without a single gore or gusset. Instead, therefore, of presenting sharp angles which cut in upon the sides, and which either tear the skin or are themselves torn up, these portions, supported by slender bones, or thin steels where necessary, curve gently and smoothly over every corresponding curve of the chest, and at once exhibit all the beauty of the most perfect form, and yield the most perfect comfort. With great propriety, also, the bones at the lower and lateral part of the stays are all thrown either before or behind the hanch, so that upon the side, above the hanch, there is no bone to bend by the heat of the body, to form itself into an angle, and to excoriate the side, as in all other stays. Mrs. Geary's stays, constructed on these principles, are preservative at once of health and beauty, and are indispensable to dress, which give a perfect idea of their advantages."

Seriously, Mr. Coulson's book is lively, well written, and indicative of much ingenuity and practical information. We have no doubt that it will become extremely popular.

---

Aneurism of the Arteria Innominata. By Sir David J. H. Dickson, M.D. Physician to the Royal Naval Hospital, Plymouth.

The following case, in detail, was read at the meeting of the British Association in Bristol last year. We have been favoured with an abstract of it by a friend.

A marine, aged 40, was received into the Plymouth Hospital, on the 5th of April, 1835, with indomitable cough, wheezing respiration, frothy expectoration, occasionally muco-purulent and scanty. He complained of tightness rather than pain, with oppression under the upper part of the sternum. The pulse was variable, being generally irregular. The breathing was loud and laborious, with mucous, sibilous, and sonorous rattle occasionally—the sibilous being the most predominant in the *exacerbations*, which assumed all the characters of asthma. At first it was supposed that there was chronic bronchitis, with thickening and narrowing of the air-tubes, and dilatation of the heart; but it was soon concluded that some tumor pressed upon the trachea. The weather and remedies had much influence over the symptoms at first, but gradually the relief was less marked —the dyspnœa became more urgent—the lips and face more livid—and the patient in a state bordering on asphyxia. He died on the 2d of June, 1836.

On removing the sternum a tumour, larger than an orange, presented itself, and proved to be an aneurism of the arteria innominata engaging the whole extent of that trunk. Pressure had rendered the left vena innominata impervious, and the blood had much difficulty in returning from the right vein—thus accounting for the turgid state of the vessels of the head and neck. The trachea was pressed upon and adherent to the tumour. The aorta, previously to giving off any of its principal branches, was enormously enlarged, and its coats unequally thickened, but without any deposits. A second aneurism, the size of a walnut, was seen appended to the artery, below the obliterated ductus arteriosus, by a narrow neck. The whole of the thoracic aorta was in a state of aneurismal dilatation. The aneurismal parts were nearly filled with concentric layers of coagulated blood, the most external being deprived of colouring matter, and intimately attached to the inner coats of the vessels. The rings of the trachea had resisted the pressure of the tumor, but the coats, and the inter-annular and mucous membranes were absorbed. The coats of the thoracic aorta throughout were thickened, softened, and deprived of elasticity. There was much frothy mucus in the bronchi, and the heart was larger than natural. The lungs were not diseased.

---

Extract from the Charter of the New London University.

The following passages embrace all the points of interest to the public, divested of technical forms and verbiage.

officers, &c. &c.

Our Right Trusty and Right Well-beloved Cousin William Cavendish Earl of Burlington,

The Right Reverend Father in God Edward Lord Bishop of Durham,

The Right Reverend Father in God William Lord Bishop of Chichester,

Our Right Trusty and Well-beloved Councillor Henry Baron Brougham and Vaux, and

Our Trusty and Well-beloved George Biddle Airy, Esq., our Astronomer Royal, and Fellow of the Royal Society.

Andrew Amos, Esq., Barrister at Law,

Thomas Arnold, Doctor in Divinity,

John Austin, Esq., Barrister at Law,

Neil Arnott, Esq., Doctor in Medicine,

John Bacot, Esq.,

Francis Beaufort, Esq., Captain in our Royal Navy, Hydrographer of the Admiralty, and Fellow of the Royal Society,

Archibald Billing, Esq., Doctor in Medicine, and Fellow of the Royal College of Physicians,

William Thomas Brande, Esq., Vice-President of the Royal Society,

James Clarke, Esq., Doctor in Medicine, Fellow of the College of Physicians, and of the Royal Society,

Philip Cecil Crampton, Esq., Doctor of Civil Law, Fellow of the Royal Society, and Our Surgeon-General in Ireland,

John Dalton, Esq., Doctor of Civil Law, and Fellow of the Royal Society,

William Empson, Esq., Barrister at Law, Professor of General Polity and the Laws of England at the East India College,

Michael Faraday, Esq., Doctor of Civil Law, Fellow of the Royal Society,

Sir Stephen Love Hammick, Bart., Doctor in Medicine, Fellow of the Royal College of Physicians, and Fellow of the Royal Society,

John Stevens Henslow, Clerk, Master of Arts, Professor of Botany in the University of Cambridge,

Cornelius Hewett, Esq., Doctor in Medicine, and Downing Professor of Medicine in the University of Cambridge,

Thomas Hodgkin, Esq., Doctor in Medicine,

Francis Kiernan, Esq.,

John George Shaw Lefevre, Esq., Fellow of the Royal Society,

John William Lubbock, Esq., Vice-President and Treasurer of the Royal Society,

Sir James M'Grigor, Baronet, Doctor in Medicine, Doctor of Civil Law, Fellow of the Royal Society, Fellow of the College of Physicians, one of our Physicians Extraordinary, and Director-General of the Army Medical Board,

Richard Rainy Pennington, Esq.,

Jones Quain, Esq., Doctor in Medicine,

John Rideout, Esq.,

Peter Mark Roget, Esq., Doctor in Medicine, Secretary of the Royal Society,

Nassau William Senior, Esq., one of the Masters of Our High Court of Chancery, and Fellow of the Royal Society,

Joseph Henry Jerrard, Doctor of Laws, Principal of the Bristol College,

Richard Sheepshanks, Clerk, Fellow of the Royal Society,

John Sims, Esq., Doctor in Medicine,

Cornop Thirlwall, Clerk, Fellow of Trinity College, Cambridge,

James Walker, Esq., Fellow of the Royal Society, and

Henry Warburton, Esq., Member of the Commons' House of Parliament, and Fellow of the Royal Society.

That the Chancellor, Vice-Chancellor, and Fellows for the time being, shall have the entire management of, and superintendence over the affairs, concerns, and property of the said University; and in all cases unprovided for by this our charter, it shall be lawful for the Chancellor, Vice-Chancellor, and Fellows, to act in such manner as shall appear to them best calculated to promote the purposes intended by the said University; and the said Chancellor, Vice-Chancellor, and Fellows, shall have full power from time to time to make, and also to alter, any by-laws and regulations (so as the same be not repugnant to the laws of our realm, or to the general objects and provisions of this our charter), touching the examinations for degrees and the granting of the same, and touching the mode and time of convening the meetings of the Chancellor, Vice-Chancellor, and Fellows, and in general touching all other matters whatsoever regarding the said University; and all such by-laws and regulations, when reduced into writing, and after the common seal of the said University shall have been affixed thereto, shall be binding upon all persons members thereof, and all candidates for degrees to be conferred by the same, all such by-laws and regulations having been first submitted to one of our principal Secretaries of State, and approved of, and countersigned by him.

That once, at least, in every year, the said Chancellor, Vice-Chancellor, and Fellows, shall cause to be held an examination of candidates for degrees; and on every such examination the candidates shall be examined, either by examiners appointed for the purpose from among the Fellows, by the said Chancellor, Vice-Chancellor, and Fellows, or by other examiners so to be appointed; and that on every such examination the candidates shall be examined in as many branches of general knowledge as the said Chancellor, Vice-Chancellor, and Fellows, shall consider the most fitting subjects of such examination. And whereas it is expedient to extend the benefits of colleges and establishments already instituted, or which may be hereafter instituted, for the promotion of literature, science, and art, whether incorporated or not incorporated, by connecting them for such purposes with the University created by this our royal charter, We do hereby further will and ordain that all persons shall be admitted as candidates for the respective degrees of Bachelor of Arts, Master of Arts, Bachelor of Laws, or Doctor of Laws, to be conferred by the said University of London, on presenting to the said Chancellor, Vice-Chancellor, and Fellows, a certificate from any of the institutions hereinafter mentioned, to the effect that such candidate has completed the course of instruction which the said Chancellor, Vice-Chancellor, and Fellows, by regulation in that behalf shall determine.

That such certificates as aforesaid may be granted from our college called University College, or from our college called King's College, both situate in London, or from such other institution, corporate, or unincorporate, as now is, or hereafter shall be, established for the purposes of education, whether in the metropolis or elsewhere within our United Kingdom, and as we, under our sign manual, shall hereafter authorise to issue such certificates.

And for the purpose of granting the degrees of Bachelor of Medicine, and Doctor of Medicine, *and for the improvement of medical education in all its branches, as well in medicine as in surgery, midwifery, and pharmacy:*—We do further hereby will and ordain that the said Chancellor, Vice-Chancellor, and Fellows, shall, from time to time, report to one of our principal Secretaries of State, what appear to them to be the medical institutions and schools, whether corporate or unincorporated, in this our metropolis, or in other parts of our United Kingdom, from which, either singly or jointly with other medical institutions and schools in the country, or in foreign parts, it may be fit and expedient, in the judgment of the said Chancellor, Vice-Chancellor, and Fellows, to admit candidates for medical degrees, and on approval of such report by our said Secretary of State, shall admit all persons as candidates for the respective degrees of Bachelor of Medicine, and Doctor of Medicine, to be conferred by the said University, on presenting to the said Chancellor, Vice-Chancellor, and Fellows, a certificate from any such institution or school, to the effect that such candidate has completed the course of instruction which the said Chancellor, Vice-Chancellor, and Fellows, by regulation in that behalf shall determine; and it shall be lawful for the said Chancellor, Vice-Chancellor, and Fellows, from time to time, with the approval of one of our principal Secretaries of State, to vary, alter, and amend any such reports, by striking out any of the said institutions or schools included therein, or by adding others thereunto.

That the said Chancellor, Vice-Chancellor, and Fellows, shall have power, after examination, to confer the several degrees of Bachelor of Arts, Master of Arts, Bachelor of Laws, Doctor of Laws, *Bachelor of Medicine, Doctor of Medicine, and to examine for medical degrees in the four branches of medicine, surgery, midwifery, and pharmacy,* and that such reasonable fees shall be charged for the degrees so conferred as the said Chancellor, Vice-Chancellor, and Fellows, with the approbation of the Commissioners of our Treasury, shall from time to time direct; and such fees shall be carried to one general fee fund for the payment of the expences of the said University, under the directions and regulations of the Commissioners of our Treasury, to whom the accounts of income and expenditure of the said University shall once in every year be submitted, which accounts shall be subject to such examination and audit as the said Commissioners may direct.

That at the conclusion of every examination of the candidates, the examiners shall declare the name of every candidate whom they shall have deemed to be entitled to any of the said degrees, and the departments of knowledge in which his proficiency shall have been evinced, and also his proficiency in relation to that of other candidates, and he shall receive from the said Chancellor a certificate, under the seal of the said University of London, and signed by the said Chancellor, in which the particulars so declared shall be stated.

Provided always, that all by-laws and regulations made from time to time touching the examinations of candidates, and granting of degrees, shall be submitted for the consideration of one of our principal Secretaries of State, to be approved of by him.

---

Ordinance of the College of Surgeons on the Recognition of Teachers.

The slovenly manner in which every thing relative to public instruction is managed in this country, has long been a subject of astonishment to intelligent foreigners, and of regret to the thinking portion of ourselves. For aught that our public bodies can tell, teachers may be more ignorant than pupils, and there is no efficient guarantee for their possession of an adequate amount and kind of information.

They "manage these things," and many others, "better in France."—There, normal schools are established for the education of teachers, and the latter are not allowed to commence the business of instruction, until they have submitted to a formal examination, and have obtained the credentials of qualification for their office.

This is a plan so consistent with the rules of common sense, that it is wonderful that any government which professes to watch over the best interests of its subjects, could neglect it. The attention of our own Legislature has been drawn to the subject, and at the present moment there is in existence in this country an Education Society, and Education Committee, comprising the most able, liberal, and philanthropical men of the day, whose professed object is to effect the establishment of normal schools and examinations for teachers.

The College of Surgeons have in this instance shewed their community of feeling with the spirit of the age, and disregarding the outcry which ignorance may excite, have published the following very proper Ordinance.

"The Council of the College, at an extraordinary meeting, on the 1st instant, established the following Ordinance, relating the recognition of the certificates of teachers of anatomy and surgery in England and Wales:—

That in future no person be recognized by this College as a teacher of anatomy, physiology, and pathology, or in surgery, in England and Wales, until he shall have undergone an examination before the Council of the College on two separate days. The first examination to be in anatomy and physiology; the second in pathology, and on the principles and practice of surgery.

That no fee be demanded for these examinations; and that the recognition of the College be conveyed in the usual form of a letter from the Secretary.

By order,

Edmund Belfour, *Sec.*

November, 18, 1836."

It is evident that this Ordinance is prospective only. It says that *in future* no teacher shall be *recognized,* except in compliance with its enactments. The act of recognition, as every body concerned in teaching knows, is a summary and final one. A teacher applies to the College for the recognition. The College assents or refuses, and if it assents there is an end of the matter. All who are at present teachers have been recognized, and of course, and properly, the Ordinance does not affect them.

The only rational objection that can be urged against this measure, is one that deserves the most serious consideration. It may be said that the Council consists of those who are teachers now, and that they ought not to be judges in cases of men who must be their friends or rivals. A very proper and unanswerable observation. It is quite clear that either no teacher should be a member of the Council, or that the Council should not be an examining body. The College had best look to this, for if existing teachers are to decide on the competency of future ones, this otherwise judicious regulation will end only in failure and disgrace.

---

### Parisian Medical Club.

When we wrote our remarks on the probable, or at least possible, consequences of these institutions in this country, we had no idea that they were assuming such a magnitude among our Gallic neighbours. A club, on a large scale, is in the course of organization in Paris, embracing the names of Marjolin, Lisfranc, Amussat, Jul. Cloquet, Magendie, Biett, Bouillaud, Rostan, Esquirol, and many others!! The members are to pay 22 francs annually, in return for which they are guaranteed medical and surgical advice and attendance—medicines—and even nurses! All operations, of whatever nature, are included. Even the teeth are to be operated on, renewed, &c. for the annual sum of less than a pound sterling! If this be not a "sign of the times," we confess that we are blind to signals. Our Dublin contemporary consoles himself with the reflection that "mankind value most what costs them most." This may be the case; but then there are more than one set of ideas in the minds of men. Phrenologists have discovered organs in the brain which counteract other organs, or neutralize them. So there are such things as avarice, economy, poverty, &c. which may very much limit the above-mentioned principle of our sanguine contemporary. We suspect that things are not valued much *merely* because they cost much money, but because articles are generally dear that are good. One physician's advice costs the same as that of another—and yet we see people prefer giving the fee to some in preference of others. We every hour see opulent people running over the town in search of cheap bargains. How does this quadrate with the principle quoted? It is of very little consequence how the article is *valued*, after it is purchased. It is the *purchase* of the cheap article, and not the estimation of it subsequently, that affects the market.

---

### Caries of the Bones of the Elbow Joint Cured by Excision. By James Edward, M.D. of Forfar.

Peter Crammond, labourer, aged 18 years, applied to me about sixteen months ago, complaining of acute pain in the elbow-joint, for which he could assign no reason. In examining it, the slightest motion increased the pain, and the surrounding integuments were swollen, but retained their natural colour. The arm was a little emaciated, both above and below the joint, but no fluctuation could be detected, and all the remedies that were applied had but little effect. About nine months after he became my patient, the pain and swelling increased, and the surrounding integuments lost their natural colour; now, also, a distinct fluctuation could be perceived at the inner side of the olecranon. By making an incision through the integuments into the abscess, and by introducing a probe, caries of the heads of the bones could be felt. A few weeks after this, fluctuation could also be distinguished over the head of the radius; when the abscess was opened, fœtid matter issued, and, by probing, caries of the bone could be discovered. A great many remedies had now been applied by different medical men, but all without effect, and on going to the Dundee Infirmary, amputation was there proposed, to which the patient would not consent. About six weeks ago, after his health had suffered severely, his pulse being quick, his appetite greatly impaired, and profuse sweats occurring occasionally, I prevailed on him to allow me to remove the heads of bones of the joint. I performed the operation in the following manner.

After placing the patient on his face on a table, an assistant kept the arm in a proper position, and then, care being taken to avoid the ulnar nerve, a transverse incision was made into the joint close above the olecranon, extending from the inner side of the process to the external tuberosity of the humerus. Next, an incision about two inches in length, was made upwards and downwards, in the longitudinal direction of the arm, forming two square flaps, and giving the wound (as is recommended by Professor Syme), the resemblance of the letter H. The flaps were next detached from the parts below them; the olecranon and sigmoid cavity of the ulna, and the head of the radius were taken off by Liston's bone forceps. The ligaments and soft parts around the lower end of the humerus were carefully separated from the bone for about two inches upward. The extremity of the humerus was then sawed off beyond the tuberosities. The patient lost but little blood in the operation, which lasted for three quarters of an hour, and a few small bloodvessels only required to be secured by ligatures. The edges of the wound were brought together by adhesive plaster, compress and bandage, and the arm was placed in a sling in a semibent position. Smart febrile excitement supervened for a few days after the operation, but by cooling lotions to the arm, purgatives, opium and calomel, this excitement was soon abated, and his strength was gradually restored by a nourishing diet and gentle exercise. The wound has now closed, and he can turn his arm, which is about two inches shorter than the other, inward and outward, and suspend a heavy weight, and also raise it to a considerable distance. On examining the excised parts of the bones, the head of the ulna, radius, and humerus, were very carious and diseased. The articular cartilages were ulcerated in several parts towards the synovial surface, and were softened and almost detached from the extremities of the bones. This operation, in my opinion, is a severe shock to the system, but I am convinced there are very few patients labouring under such a malady, who would not rather submit to it, severe as it is, than to amputation. The arm, with all its deficiencies, is already far better than any artificial substitute ever yet invented.

The preceding case is highly creditable to Dr. Edwards. We insert it in order to shew, what is indeed unnecessary, the diffusion of sound scientific principles, and of practical attainments among our provincial brethren.

---

How to become a Crack Physician, 150 Years ago. By Dr. Mandeville.

" Where shall you find a physician now-a-days, who makes that stay with his patients, which it is plain the ancients must have done to make the noble prognosticks we have from them. But this would not only be too laborious, but a tedious way of getting money: self-interest now gives better lessons to young physicians. If you are not extraordinary in any of the branches I have named, rather than that you should spend your time before the squalid beds of poor patients, and bear with the unsavory smells of a crowded hospital, show yourself a scholar, write a poem, either a good one, or a large one, compose a Latin oration, or do but translate something out of that language with your name to it. If you can do none of all these, marry into a good family, and your relations will help you into practice: or else cringe and make your court to half a dozen noted apothecaries, promise 'em to prescribe loads of physic, never to forget the melodious sound of *bolus*, and always to make your bills like the chimes of the Exchange—ring with a " repetatur tertia quaque hora :" nay, get but in favour with one that has great business, and your's is done. Otherwise be a rigid party-man; it is all one Whig or Tory, so you are but violent enough of either side;—or, if you can chat, and be a good companion, you may drink yourself into practice; but if you are too dull for what I have hitherto named, and in reality good for nothing, you must say little, and be civil to all the world—keep a set of coffee-houses, observe your certain hours, and take care you are often sent for where you are, and asked for where you are not; but though in them you are forced to sit idle and loiter away your time all day long, yet out of 'em always counterfeit a man that is in haste, and wanted in a great many places. As for the rest, study what Demea said of his brother, to be clemens, placidus, nullios lædere, arridere omnibus; contradict nobody, never open your lips without a smile, and give no peace to your hat."

### Provincial Medical Journal.

A new periodical of this description is about to appear in Ireland, under the editorial management of Mr. Phelan, already well known to the profession. We think it likely to succeed. It is to be conducted on what may be termed liberal or popular principles, and with an especial eye to medical reform. It is true that a provincial medical journal never did, and probably never will, succeed in England. The reason is obvious. There are always four or five metropolitan journals in existence, the great majority of which are liberal, and which journals can be circulated with far more celerity through the country, than can any journal from a provincial town. In Ireland it is very different. Only one metropolitan journal exists there—and it has a strong leaning to aristocratic principles, though conducted with great talent and honorable feeling. Still there is great room for a periodical in that country, which would take on a more popular character, and advocate freely, but temperately, reform of medical abuses. We see that there has been a public meeting in Clonmel, where pledges have been given to support a journal of the above description. We need hardly say that we wish it every success, and shall contribute to make it known abroad and at home.

### Leech Trade.

France, Hungary, and Germany are nearly exhausted of leeches, and the trade is now supplied from Wallachia and Moldavia. Depôts are established at Bucharest, whence they are brought to France in the post-carts. In 1825, France exported more than a million; and now, as above stated, is obliged to import.

Is this the effect of the doctrine physiologique? What a triumph to Broussais is this. He has created in France a new commerce, and, as is the case with all conquerors, his glory is at the expense of blood. Here is another subject for the pen of the author of the "Conquetes des Français."

### A Notable Discovery.

Most practitioners have felt the disappointment of not being able to draw blood from the veins of the arm, at times when they were most anxious to do so. A German discovery to remedy this inconvenience has been copied by most of our contemporaries. The plan is said to be "applicable to all cases where open veins do not give a sufficient quantity of blood, or in bleeding fat persons where the veins are not apparent." M. Burdach tells us that, in such cases, it is only necessary to apply a ligature to the *other arm*, when, in a few minutes, *both* arms will swell with blood, and then the ligature (which ligature?) is to be relaxed, and compression is to be made with the thumb, "that the blood may flow in a jet." Upon what physiological principle this phenomenon is to be explained we know not. We dare not deny that the moon is made of green cheese—because we cannot disprove the assertion. But we are strongly disposed to the truth of this discovery, and for a reason which must come home to the memory of every man of experience—namely, that we have all seen *both arms tied up*, and the phlebotomist digging into every vein, visible or invisible, without success. The practice of applying ligatures to both arms and both legs, to stop an ague fit, had something to ground a *rationale* on, because, when the round of the circulation was materially narrowed, it might be expected that the current would be augmented in the circumscribed channels, and the cold fit of ague shortened. But this piece of transcendentalism transcends even the imagination, and leaves us in the comfortable creed of the Italian—"credo quia impossible."

### Dr. Bostock's Erratum.

Sir,—In the last Number of your Journal, you have inserted an analysis of mine, as an appendage to Mr. Coulson's paper; there is in it a typographical error, which somewhat affects the sense, or at least the correctness of the description. It is the last word of the second paragraph, which is printed "urine," but which ought to be "urea"—an error which, I doubt not, is attributable to my bad writing. Perhaps you would not object to stating it as an erratum in your next Number, or at the end of your volume.

I hope you will pardon me for giving you this trouble, and believe me,

Your most obedient,

*Upper Bedford-pl.* J. Bostock.

*Oct.* 14*th*, 1836.

---

### Phlebitis.

Mr. Harrold, of Cheshunt, writes to us that, when attending the London Hospital in 1790-1, phlebitis was found to be of very frequent occurrence, and the cause was at length traced to the use of soiled sponges, applied to the incisions to cleanse them before the bandage was applied. Sir Wm. Blizard gave an order that no such sponges should be employed, and phlebitis disappeared.

---

### Obituary.

We regret to record the death of a very able medical practitioner, Mr. Sheppard, of Witney—a half-pay naval surgeon, and a man of excellent abilities. He was lately appointed by the Admiralty to revise and collate the medical journals of the navy, in order that a report might be prepared on the state of health in our ships of war, and embracing various other topics of interest. He had scarcely entered on his new avocation, however, before he experienced an apoplectic seizure, while writing at his desk in Somerset-place. From this he recovered, and went down to Witney, where he had a relapse, and died in the course of an hour. He was the author of a great part of the Eclectic Article on Yellow Fever in Dr. Johnson's third and subsequent editions of his work on Tropical Climates. He has left two children, now bereaved of both parents, to mourn their irreparable loss.

---

